# Retrusive Chin Reconstruction After Burn Injuries Using Submental and Labiomental Fat Flaps: An Innovative Method

**Published:** 2009-11-23

**Authors:** Ali Manafi, Aydin H. Pooli, Peiman Habibollahi, Lila Saidian

**Affiliations:** ^a^ Hazrat Fatemeh Hospital, Iran University of Medical Sciences, Tehran, Iran; ^b^ Cedars-Sinai Medical Center, Los Angeles, California; ^c^ Tehran University of Medical Sciences, Tehran, Iran; ^d^ University of California, Los Angeles (UCLA), California

## Abstract

**Objective:** A simple method has been introduced for augmentation and restoration of normal chin protrusion as an important element of facial contour in patients suffering from severe skin retraction and scar resulting from head and neck area burns. **Patients and methods:** For releasing skin retraction and compression of the mental area subcutaneous fat tissues, under general anesthesia, 2 incisions were made in the submental and labiomental areas, each about 3 to 5 cm. After dissection of the superior portions of labiomental and inferior pedicle of submental fat pads, turning over and attaching them together, the skin over the area was reconstructed as needed. **Results:** Thirty-four patients with pseudomicrogenia were involved. In 32 patients, the procedure was performed successfully and the results were evaluated as excellent in 15 cases and good in 17 cases. However, in 2 patients, suboptimal results were obtained, which were corrected using genioplasty in one of them. The other one did not consent for any further surgeries. Split-thickness skin grafting was performed in 3 cases. Tissue expansion and full-thickness skin grafting were used in 3 others for resurfacing the area. **Conclusion:** Taken together, the current technique might be helpful in restoring normal chin protrusion and can be used besides available methods for resurfacing and reconstruction of the defective skin for enhancing the facial appearance.

Maxillofacial skeletal deformities following burns of the face and neck area are far more common in practice than the paucity of reported cases would indicate.^1^ Wound healing and skin graft contraction in the lower lip and chin area frequently produce eversion and ectropion of the lower lip presenting as a protrusion of rolled-down lip vermilion and mucosa. Blunting of the sulcus between the lip and the chin adds to deformity and can make the chin appear small.^2^ Associated neck contracture can deteriorate the pseudomicrogenia by obscuring the cervicomental line.^2^

As an important visual component of the face, chin bears noticeable functional importance. The vermilion constituent of the lip, various muscle layers, and the bony gonion as different tissue constituents of chin besides its numerous convexities and concavities^3^ make reconstruction of the chin very challenging.^4^,^5^

Genioplasty and plastic allografts are among the used methods for chin augmentation. Like any other surgical procedure, these procedures can cause complications such as edema, infection, and bleeding. The most common complications associated with genioplasty would include potential for relapse, neurologic deficits, and soft tissue deformities. Bone erosion and implant displacement and extrusion are also reported with the use of implants,^6^ although developing new techniques for chin reconstruction is of utmost importance and should be aimed toward simple techniques with lower complications.

We have examined a new technique for chin augmentation and reconstructing its protrusion, without the use of implants or osteotomy in our hospital on a series of patients. Here, we try to describe the method, its results, and complications associated with it during the follow-up.

## MATERIALS AND METHODS

The study was performed in Iran University of Medical Sciences. Between 1997 and 2008, patients suffering from burn injury of the head and neck area who completed the written informed consent were involved in the study. All patients had scar contracture in the neck and submental area that had caused severe pseudomicrogenia.

The study process was described for the patients in detail. All patients were informed about possible complications and failure of the surgery after which they filled a written informed consent to be involved in the study and all medical procedures were performed without any charge for the patients. Demographic data on age, sex, profession, burn injury, and grade of the preliminary burn lesion were collected using a simple questionnaire.

## SURGICAL PROCEDURE

**Figure 1:**
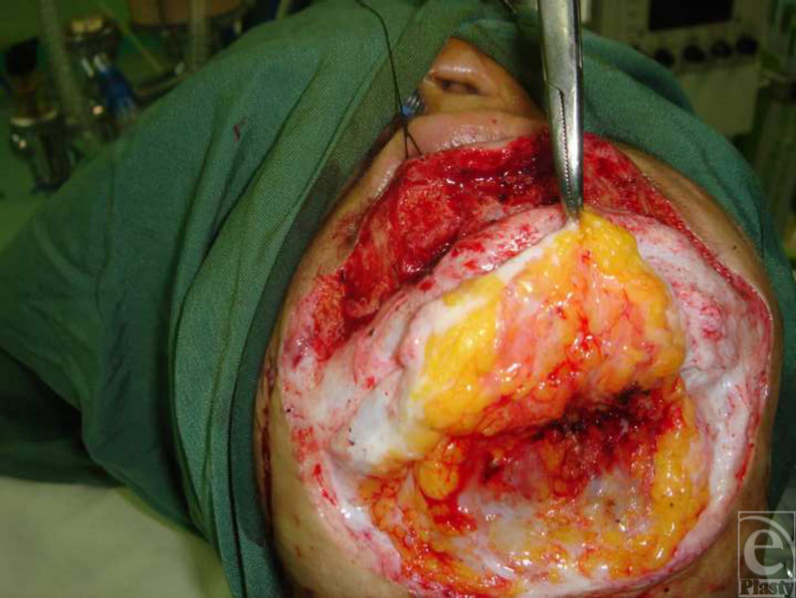
Submental fat pad flap with superior pedicle (frontal view).

**Figure 2:**
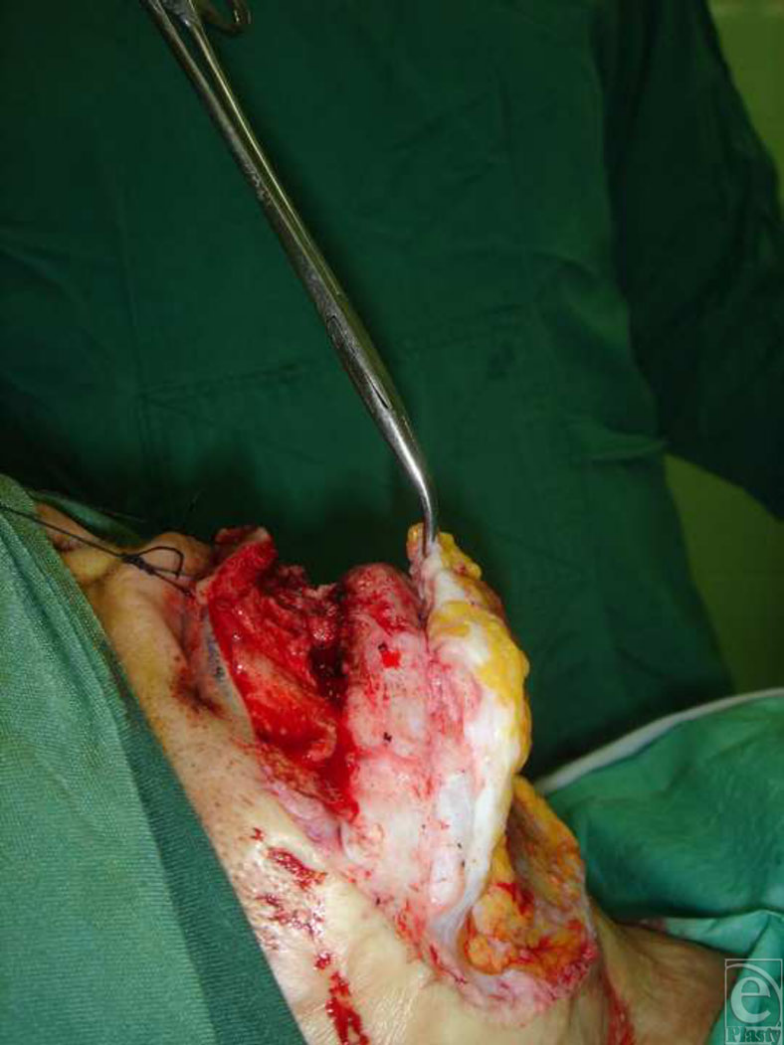
Submental fat pad flap with superior pedicle (lateral view).

After general anesthesia and preparation of the area, an incision of about 3 to 4 cm was made on labiomental fold. At the submental area, the same cut was incised. After mobilization of the skin flap, labiomental fat pat at the inferior pedicle was dissected. The same method was applied in the submental incision and submental fat pad at the superior pedicle was dissected and mobilized like hinge flap (Figs 1 and 2). Then both flaps were turned over and stitched together at the place of chin protrusion. Finally skin reconstruction was performed as indicated.

**Figure 3:**
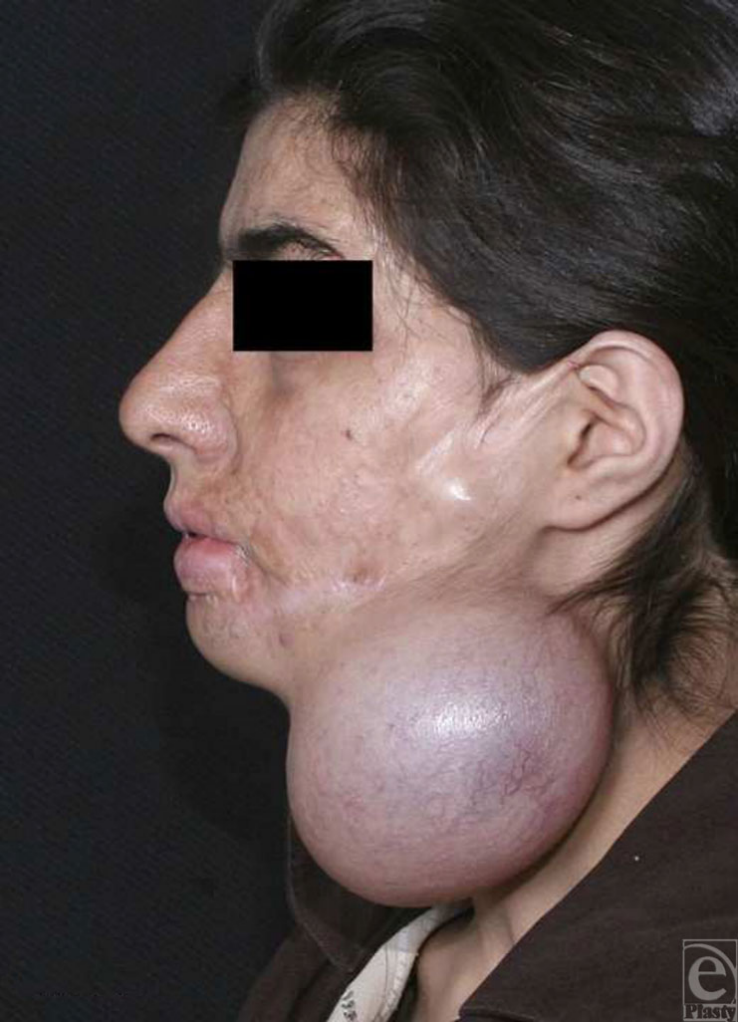
Tissue expansion was used for patients with inadequate or improper skin in chin area.

In case of inappropriate overlying skin, the scarred skin was removed and split-thickness skin was grafted to the chin and neck area, or using tissue expander at the neck region (Fig 3), flaps were turned and attached to the scary part. Genioplasty and alloplastic chin augmentation were also considered for cases in which the reconstruction failed to create the appropriate chin protrusion.

## RESULTS

From 1997 up to 2007, 34 patients accepted to enter our study. Twenty-seven cases were females and the rest were males. Patients' age was in the range of 19–48 years with the mean of 28.4 years. All patients were suffering from inferior lip, chin, and neck burn scars with or without involvement of other areas. Chin appearance in almost all patients was pseudomicrogenia, which was due to the compression of chin fat pad resulting from burn scar. In 25 cases, burn was caused by flame, in 6 cases by hot liquids, and in the rest of the cases (3 patients) due to suicide with inflammable liquids such as gas oil.

In 31 cases, after making the necessary incisions, submental fat pads were dissected and stitched according to the described method. Afterward, the incisions were repaired. In 3 cases due to inappropriate skin of the chin area, split-thickness skin grafting was performed after removing the scarred skin over the chin. For the rest of the patients (3 patients), after expansion of the skin in the neck area, which was planned prior to the surgery, the skin flap was turned over the chin area and replaced instead of the unsuitable scary skin.

Eyelid ectropion, lower lip ectropion, and microstomy repair were done in 8 cases, and in 18 patients other surgeries such as nose or other facial elements reconstruction were done. All patients were discharged after 24 to 96 hours.

Only in a few cases, surgical complications occurred. Infection occurred at the labiomental incision in one patient, and in 2 cases, unilateral swelling was seen because of small hematoma that was treated with small incision and drainage of the hematoma. Most complications were due to skin grafting and in 2 out of 3 patients, patchy incomplete graft taking was observed.

Surgical outcome was quite good in almost all the patients. The results were estimated to be good and excellent in 17 and 15 patients, respectively, but in 2 cases, outcome was not satisfactory. Genioplasty about 6 mm was performed in one of the patients whose result was not acceptable but the other patient was satisfied with the result and requested for no further surgeries.

All of the patients were satisfied with the result of the surgery but the one for whom genioplasty was performed. Most patients stated that their self-confidence and appearance have been improved after the surgery, and 7 patients claimed that if there is any surgical method for skin augmentation over the area, they wish to undertake it.

## REPRESENTATIVE CASES

### Case 1

**Figure 4:**
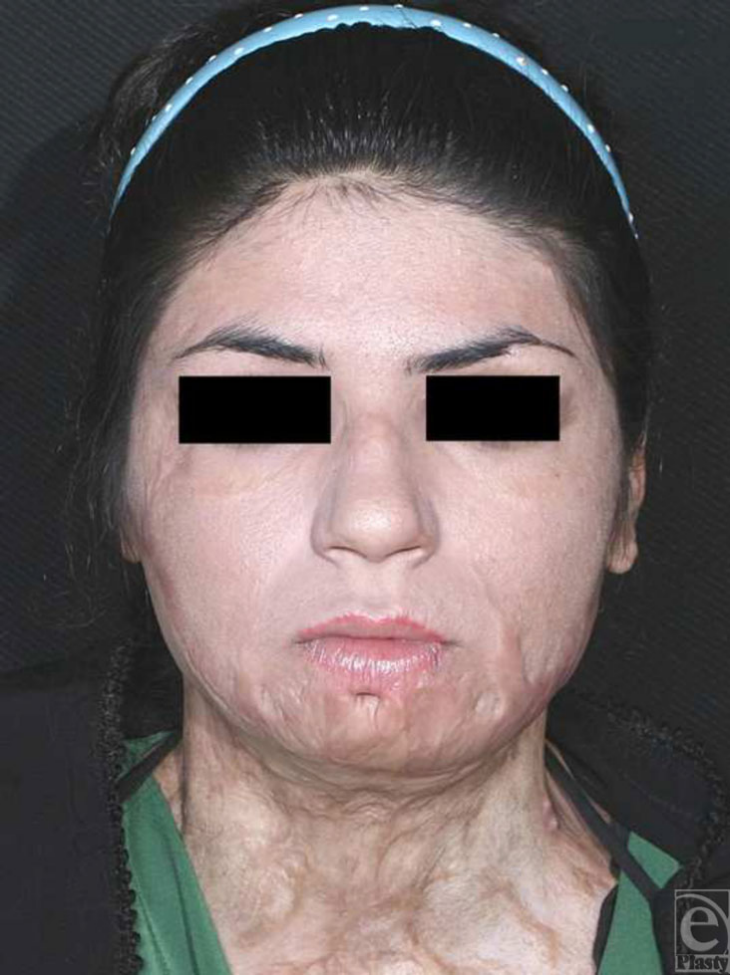
Preoperative frontal view.

**Figure 5:**
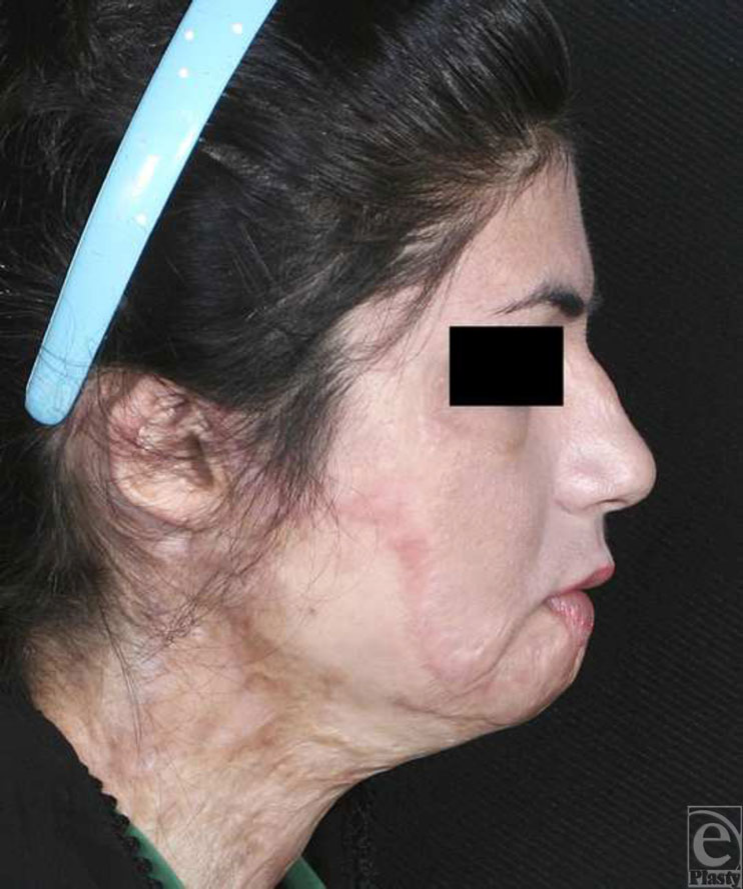
Preoperative lateral view. Deformity of lower lip, chin retrusion, and unidentifiable chin folds besides scar of previous burn before surgery are evident.

**Figure 6:**
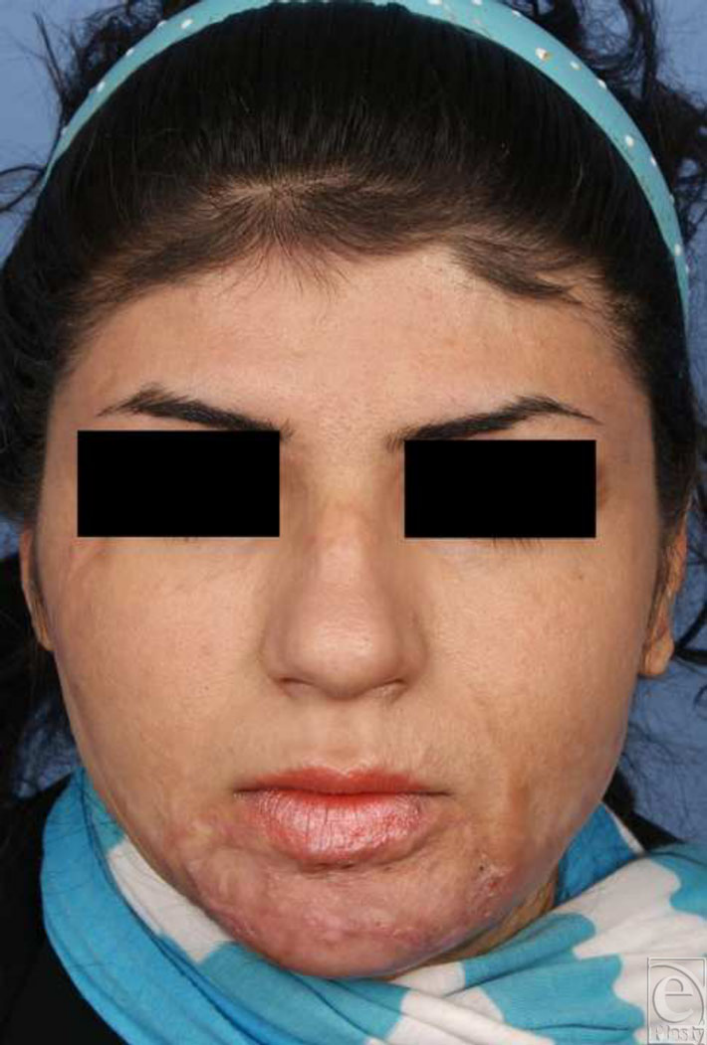
Postoperative frontal view.

**Figure 7:**
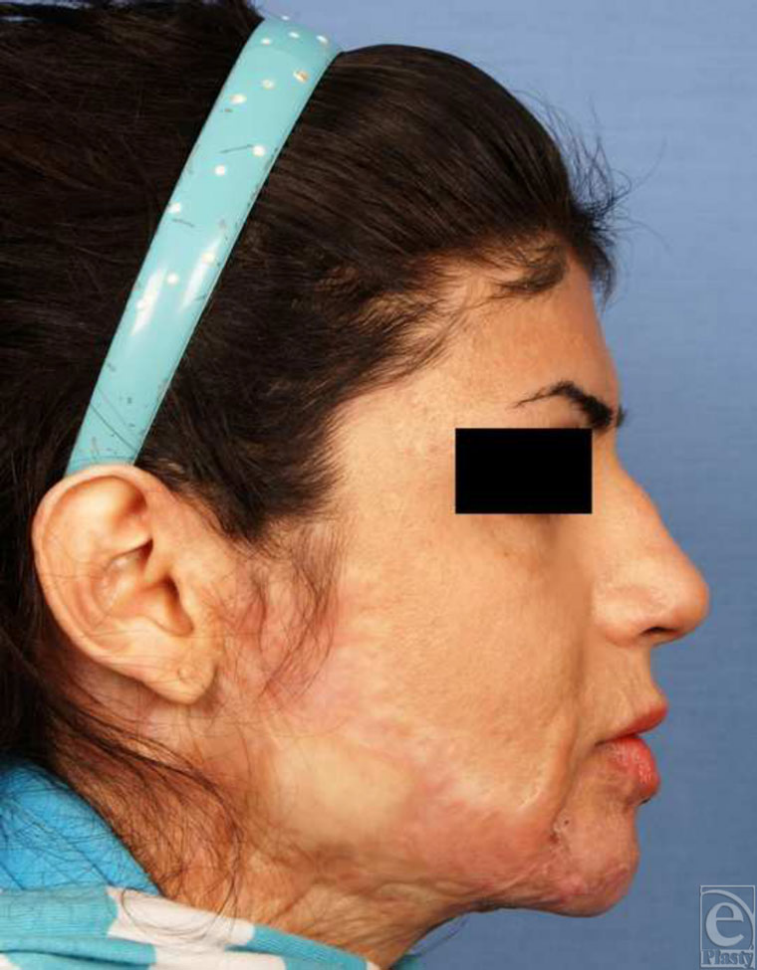
Postoperative lateral view. After surgical correction, the form of the chin is augmented significantly and labiomental and submental folds are notable.

A 24-year-old unmarried woman from Booshehr (located at northern coasts of Persian Gulf, Iran) was referred to our hospital because of disfigurement of lower lip and chin and loss of labiomental and submental folds resulted from extensive burn (caused by flame) of lower face, neck, upper trunk, and extremities at the age of 4. Under generalized anesthesia, as a result of severe scarring lower lip and chin, sacrectomy was carried out. Afterward, displaced tissues that had obliterated the natural chin folds were unroofed and by 2 hinge flaps, made of submental and labiomental subcutaneous fat pads, chin projection and folds were augmented. After sufficient homeostasis, split-thickness skin grafting from left lateral thigh was done over the chin and lower lip damaged skin and fixed by the means of tie over dressing. In the follow-up, graft taking was acceptable and appearance of lower lip and chin was augmented significantly, which resulted in patient satisfaction. Figures 4 to 7 show the pictures of the patient before and after the surgery.

## Case 2

**Figure 8:**
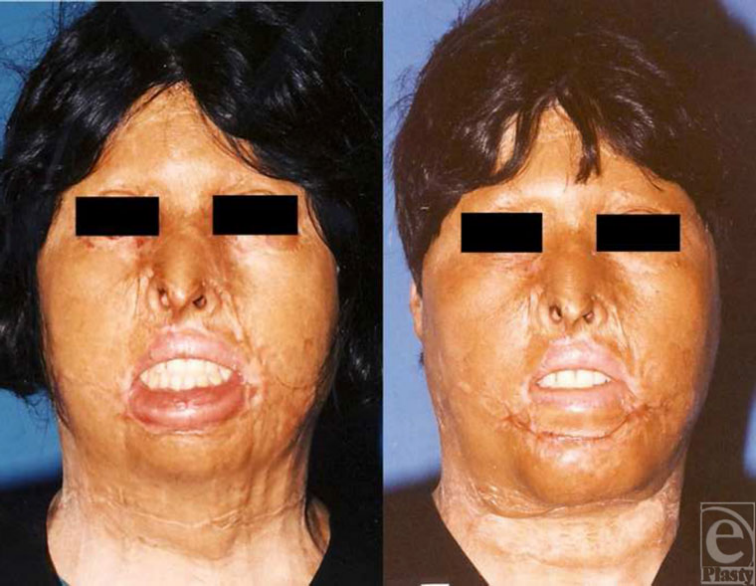
Preoperative and postoperative frontal views.

**Figure 9:**
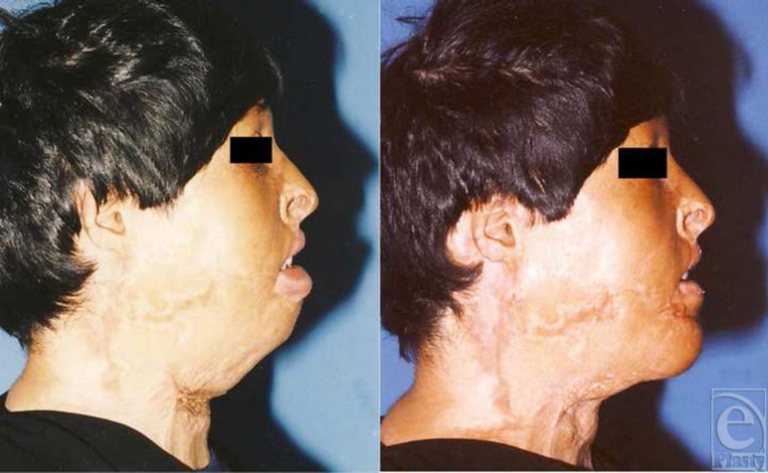
Result of the reconstructive surgery after 2 years. Severe chin retrusion in addition to previous skin grafting signs is obvious and again natural foldings of chin area are not identifiable (left). Reconstruction of the chin using subcutaneous fat has enhanced chin protrusion, appearance, and its normal folds, noticeably (right).

Another female patient at the age of 45 years was referred to the same center because of several deformities and misshapen scars in face, neck, trunk, and upper extremities caused by gas explosion and burning clothes 6 years before. Several surgeries and split-thickness grafting had been performed for correcting the deformities but still several deformities such as incomplete closure of eyelids, upper eyelids ectropion, lip incompetence, retrusive chin, and unidentifiable chin folds existed on her face. Under generalized anesthesia, upper eyelids ectropion was corrected by skin grafting and liberating the skin contracture, which was followed by chin and lower lip reconstruction. Using 2 horizontal incisions in submental area and lower lip vermilion edge about 4 to 5 cm and liberating vermilion edge and orbicularis oculi muscle from adhesions, severe ectropion of lower lip was corrected. Afterward, lower lip was suspended on the face by a strip of fascia lata (0.5 × 2 cm). Chin skin was released from underlying tissue via undermining the skin from the incisions and hinge flaps from the subcutaneous fat pads were used for correcting proper chin protrusion by overlapping the flaps and stitching them with vicryl string. Turned flaps at labiomental and submental areas were advanced about 3 and 4 to 5 cm, respectively, and then incisions were repaired and hemovacuum drain was placed under the flap. The result of the surgery after 2 years is shown in Figures 8 and 9. The patient was extremely satisfied with the outcome and microgenia or retrogenia appearance was corrected.

## DISCUSSION

Although other facial structures such as nose are considered very important, chin composition is a central part of facial aesthetics. In suitable dimension, outline, and location, it can significantly improve the normal harmony and equilibrium of the face. The chin beside the nose is a major component of facial symmetry and reflects the face general profile although, when unsuitable, it can considerably detract from an otherwise satisfying face and express unwanted features.^7^

It has been suggested that blunting of the sulcus among the lip and the chin due to burned skin contracture besides eversion and ectropion of the lower lip results in “pseudomicrogenia.” Associated contractures in the neck area usually add to the deformity,^2^ although chin augmentation should be considered as a priority in treating patients with facial burns because unsatisfying face can severely affect self-confidence and social interactions of the affected person.

Alloplastic material replacement and osteotomy are the most prevalent methods for reconstruction of the chin protrusion. Both methods have been studied for a long period and have changed over time. Other than usual complications associated with any surgical procedure, each method according to its nature has specific complications. Because of more invasiveness, chin augmentation associated with osteotomy results in more serious complications such as mandible fracture,^8^ neurologic deficits,^9^–^11^ and soft tissue deformities.^12^ Protuberant chin button^13^–^15^ and bone resorption^16^ are also reported with alloplastic chin augmentation. Inventing new techniques with simpler methods that might result in lower complications is of utmost importance.

Here, we have described a simple method for restoring normal protuberance of the chin, which can be used beside available techniques for resurfacing skin over the burned area. Lower invasiveness compared to osteotomy and the use of auto graft instead of alloplastic material are the most important advantages of the current method. Lower period of hospitalization due to its simple and contained nature might be other possible benefit.

Our series of patients showed that success rate achieved with this method is high and can be compared with other accessible methods as 33 patients accepted the outcome. Beside patient attitude, in our opinion the results were satisfactory in almost all of the patients but 2 of them in whom suboptimal results were obtained. This preliminary study reveals that further studies with larger sample size might be performed to evaluate possible complications that are not seen here and also to estimate more accurate success rate.

Taken together, the current technique might be helpful in restoring normal chin protrusion and can be used besides available methods for resurfacing and reconstructing the defective skin. More invasive approaches like osteotomy genioplasty can be considered for cases in which the current method fails to restore satisfying chin protrusion.
